# Patterns of pain and stiffness over 5 years in polymyalgia rheumatica: results from the PMR Cohort Study

**DOI:** 10.1093/rap/rkaf060

**Published:** 2025-06-05

**Authors:** Niall Hamad, Sara Muller, Samantha Hider, Richard Partington, Toby Helliwell, Henna Butt, Charles Hay, Christian D Mallen

**Affiliations:** School of Medicine, Keele University, Keele, UK; School of Medicine, Keele University, Keele, UK; School of Medicine, Keele University, Keele, UK; Midlands Partnership University Foundation Trust, Stoke-on-Trent, UK; School of Medicine, Keele University, Keele, UK; School of Medicine, Keele University, Keele, UK; Midlands Partnership University Foundation Trust, Stoke-on-Trent, UK; Histology Department, University Hospitals of the North Midlands, Stoke-on-Trent, UK; School of Medicine, Keele University, Keele, UK; School of Medicine, Keele University, Keele, UK; Midlands Partnership University Foundation Trust, Stoke-on-Trent, UK

**Keywords:** polymyalgia rheumatica, cohort study, pain, glucocorticoids, prevalence

## Abstract

**Objectives:**

To investigate the anatomical locations of pain and stiffness in people with polymyalgia rheumatica (PMR) and how these compare with the general population.

**Methods:**

A total of 739 people with PMR were invited to complete a postal survey at the time of their diagnosis. Respondents were sent further questionnaires after 1, 4, 8, 12, 18, 24 and 60 months. All questionnaires included a body manikin on which participants shaded areas of pain or stiffness lasting >1 day in the last month. The prevalence of pain was calculated in 44 mutually exclusive areas. Responses were compared with similar manikins completed at a single time point by an age- and gender-matched sample from a general population survey.

**Results:**

Completed surveys were received from 652 people with PMR at diagnosis, 244 at 24 months and 197 at 60 months. Pain was reported in a median of 16 sites at diagnosis, with the majority reporting bilateral shoulder (81%) and hip (59%) pain. After 1 month, the median number of pain areas in people with PMR was four—the same as the general population sample—but those with PMR continued to report more bilateral shoulder and hip pain. The converse was true for unilateral pain.

**Conclusion:**

Bilateral pain remains more common in people with PMR than their age- and gender-matched counterparts through the disease course. Causes of this pain could not be attributed but likely include residual disease activity, treatment sequelae and comorbidities. This knowledge will help to direct future investigations to improve quality of life for people with PMR.

Key messages

- People with PMR experience more pain/stiffness at most body sites at diagnosis than age-matched controls.
- Bilateral shoulder and hip pain are more prevalent than in controls during and after treatment.
- Our findings will guide research to understand and reduce extended glucocorticoid use for PMR.

## Introduction

PMR is a relatively common inflammatory rheumatological condition in older adults, with an estimated age-adjusted incidence rate of 95.9 per 100 000 in adults >40 years of age in the UK [1]. Classically, PMR presents with pain and stiffness in the shoulder and hip girdles, accompanied by elevated inflammatory markers (e.g. ESR and CRP) [1]. It can be severely disabling and distressing for patients, particularly when onset is sudden [2]. Once diagnosed, treatment for PMR is with low- to moderate-dose oral glucocorticoids (12.5–25 mg prednisolone daily), which is usually effective in reducing pain, stiffness and disability within days [2]. Treatment is then slowly reduced. Guidelines say this withdrawal process should take ≈18–24 months, but recent studies have shown that it is often considerably longer [3–5].

The process of making a diagnosis of PMR is not always straightforward, as it is a diagnosis of exclusion and presentation is not always classical (e.g. Mackie and Mallen [2]). This is against the backdrop of patients with an average age at diagnosis of 72 years and the multimorbidity that often accompanies this [3, 6]. As some of these morbidities may be painful (e.g. OA), it can be difficult for patients and physicians to disentangle symptoms of comorbidities from potential symptoms of PMR. Similarly, the process of tapering glucocorticoid treatment can also be problematic, with patients experiencing a resurgence of symptoms as the dose is reduced. Musculoskeletal pain is common in the age group affected by PMR [7] and it is possible that the presence of painful comorbidities that are incidentally treated by the glucocorticoid (e.g. OA, cervical spondylosis) might be making glucocorticoid reduction more difficult.

While people experiencing pain need to be helped to manage that as best they can, whatever the cause, a better understanding of the pain experienced by people with PMR over the course of their condition could help to determine when this pain should be treated with glucocorticoids and when other pain management strategies would be more appropriate.

In this article we use data from the PMR Cohort Study, an inception cohort of patients with PMR recruited from UK primary care, to investigate the anatomical locations of pain and stiffness in this condition and how these compare with the general population.

## Methods

### PMR Cohort Study

Study procedures and the baseline sample have been described in detail elsewhere [5, 8, 9]. Briefly, potential participants were identified in primary care when they were diagnosed with PMR by their general practitioner (GP) between June 2012 and June 2014. No specific diagnostic criteria were applied and participants were considered to have PMR if they received a Read code (primary care clinical coding system used the in the UK) for this in their medical record. GPs were provided with a copy of the British Society for Rheumatology (BSR) guidelines on the diagnosis of PMR [10]. Potential participants were mailed a baseline questionnaire and on return of this questionnaire received follow-up questionnaires 1, 4, 8, 12, 18 and 24 months after their initial diagnosis. All participants who had not withdrawn their consent and whose general practice agreed to take part in the study were mailed a final questionnaire in the winter of 2019–2020.

**Figure 1.**
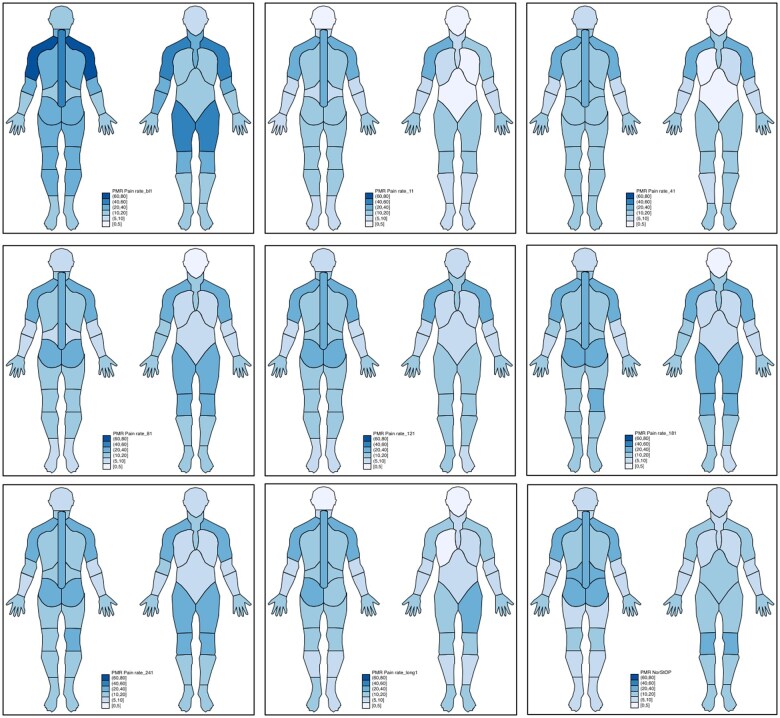
Pain prevalence—reads top to bottom, left to right over time (diagnosis, 1, 4, 8, 12, 18, 24, 60 months). Comparator cohort (random age- and gender-matched sample from the NorStOP study surveyed in 2002) bottom right.

At each time point, participants were asked to shade ‘any pain/stiffness that you had when you went to see the doctor’ (recruitment questionnaire) and ‘any recent pain/stiffness’ (follow-up questionnaires) on a full body manikin (front and back views). Separate manikins were provided for pain and for stiffness. Manikins were scored using a transparent overlay split into 44 mutually exclusive areas (Fig. 1). This method has been shown to be repeatable [11]. Data from the manikins were used to define each respondent as experiencing widespread pain [12] (spine plus at least two contralateral quadrants) and stiffness at each time point and pain/stiffness in specific regions of the body [7]. The laterality of pain in the shoulders, hands, hips and knees was also considered. Participants were also asked to rate their pain and stiffness from PMR on a 0–10 numerical rating scale (NRS), with 0 being no pain/stiffness and 10 being the worse pain/stiffness imaginable.

### The North Staffordshire Osteoarthritis Project (NorStOP) – a general population comparator

The NorStOP cohort has been described extensively elsewhere [7]. All adults ≥50 years of age from three general practices in North Staffordshire, UK were sent a postal survey questionnaire in 2001, with reminders sent to non-responders after 2 and 4 weeks. Surveys collected information on a range of health-related topics, including participants completing a blank body manikin that was assessed using the same transparent overlays as those used in the PMR Cohort Study. In the NorStOP study, participants were asked to complete only one manikin and to record any pain lasting ≥1 day in the past 4 weeks, which was described as including ‘ache, discomfort or stiffness’. A sample of 652 respondents from the NorStOP study was randomly selected to match the age and sex profile of the PMR Cohort Study respondents.

### Statistical analysis

The proportion of people with pain/stiffness in each mutually exclusive area on the manikins, with widespread pain/stiffness and with pain/stiffness in specific anatomical locations was calculated. Ratings of pain and stiffness on the NRS were highly skewed and thus were summarized as a median and interquartile range (IQR), as were the total number of pain/stiffness sites reported.

Analyses were repeated stratifying the cohort at each time point according to whether they reported use of glucocorticoids at that time.

Differences between the sexes and between age groups (<60, 60–69, 70–79, ≥80 years) and those reporting and not reporting glucocorticoids use were assessed using Kruskal–Wallis and chi-squared tests as appropriate. Groups reported use and non-use of glucocorticoids were only compared statistically from the 12-month follow-up due to small numbers reporting non-use until this point.

This study complies with the Declaration of Helsinki. Ethical approval for this study was received from the Staffordshire Research Ethics Committee (reference no. 12/WM/0021) and all patients provided written informed consent. Cell counts <5 were suppressed to ensure individual participants could not be identified.

## Results

### PMR Cohort Study—recruitment and retention

As previously reported, 739 people were referred to the study by their GP and 652 completed the baseline questionnaire and entered the cohort (adjusted response rate 90.1%). A total of 444 remained in the study at 24 months and 197 at the long-term follow-up. The mean age of the cohort at baseline was 72.4 years (s.d. 9.3) and 62.2% were female.

There was no difference in the age and gender distributions of those who entered the cohort and those who were referred but did not take part [9]. However, those who did not enter the cohort were more likely to live in neighbourhoods with higher levels of deprivation. Loss to follow-up over 2 years was associated with a range of socio-economic and health-related characteristics [5, 13].

### Pain and stiffness in people with PMR

The median number of pain sites in the PMR cohort decreased from 16 (IQR 10–24) at the time of diagnosis to 4 (IQR 0–10) at 1 month (Table 1). This remained largely flat, with a median of 5 (IQR 0–12) pain sites at the long-term follow-up.

**Table 1.** Pain characteristics over 2 years

Characteristics	Baseline (*n* = 650)	Month 1 (*n* = 599)	Month 4 (*n* = 554)	Month 8 (*n* = 527)	Month 12 (*n* = 495)	Month 18 (*n* = 470)	Month 24 (*n* = 445)	Long-term follow-up (*n* = 197)	NorStOP cohort (*n* = 652)
Pain (0–10 NRS), median (IQR)	8 (7–9)^a^	2 (1–4)^a^	2 (1–4)^a^^,^^b^	3 (1–5)^a^^,^^b^	2 (1–4)^a^	2 (1–5)^a^	2 (0–5)	2 (0–5)^a^	
Total pain sites, median (IQR)	16 (10–24)	4 (0–10)^a^^,^^b^	5 (0–11)^a^^,^^b^	5 (0–11)^a^^,^^b^	5 (0–12)^b^	6 (0–12.25)^a^^,^^b^	5 (0–13)^b^	5 (0–12)	4 (0–9)
Bilateral shoulder pain	531 (81.4)	205 (34.1)^b^	206 (37.1)^b^	181 (34.3)^b^	183 (36.8)^b^	174 (36.9)^a^^,^^b^	165 (37.0)^b^	66 (33.5)	110 (16.9)
Unilateral shoulder pain	44 (6.8)^b^	72 (12.0)^b^	74 (13.3)	66 (12.5)	61 (12.3)	66 (14.0)^b^	48 (10.8)	25 (12.7)	95 (14.6)
Bilateral hip pain	387 (59.4)^b^	123 (20.5)^b^	99 (17.8)^a^^,^^b^	117 (22.2)^a^^,^^b^	132 (26.6)^a^^,^^b^	121 (25.6)^a^^,^^b^	104 (23.3)^b^	46 (23.4)	87 (13.3)
Unilateral hip pain	43 (6.6)	58 (9.7)	60 (10.8)^a^	50 (9.5)^a^	40 (8.1)	30 (6.4)	34 (7.6)	20 (10.2)	112 (17.2)
Bilateral hand pain	173 (26.5)	72 (12.0)	76 (13.7)	84 (15.9)	81 (16.3)	85 (18.0)	74 (16.6)	37 (18.8)	91 (14.0)
Unilateral hand pain	57 (8.7)	36 (6.0)^a^	37 (6.7)^a^	38 (7.2)	37 (7.4)	29 (6.1)	27 (6.1)	16 (8.1)	52 (8.0)
Bilateral knee pain	291 (44.6)	109 (18.1)	88 (15.9)	89 (16.7)^a^^,^^b^	100 (20.1)^a^	110 (23.3)	88 (19.7)	39 (19.8)	159 (24.4)
Unilateral knee pain	68 (10.4)	52 (8.7)	42 (7.6)^a^	50 (9.5)^b^	34 (6.8)	34 (7.2)	35 (7.9)^a^	15 (7.6)	106 (16.3)
Widespread pain	364 (55.8)^b^	127 (21.1)^a^^,^^b^	106 (19.1)^a^^,^^b^	108 (20.5)^a^^,^^b^	113 (22.7)^a^^,^^b^	111 (23.5)^a^^,^^b^	101 (22.7)^a^^,^^b^	56 (28.4)^b^	133 (20.4)

Values presented as *n* (%) unless stated otherwise.

The NorStOP cohort is a random sample selected from a general population survey of people ≥ 50 years of age to match the age and gender distribution of the PMR cohort at baseline. Differences between the NorStOP and PMR cohort were not tested.

a Significant difference between males and females.

b Significant difference between age groups (<60, 60–69, 70–79, ≥80 years).

The prevalence of pain at the time of diagnosis was high in those with PMR. This was especially marked in the posterior shoulders and anterior hips (Fig. 1), with the majority of study participants reporting bilateral shoulder [*n* = 531 (81.4%)] and hip [387 (59.4%)] pain (Table 1). The majority also reported widespread pain [*n* = 364 (55.8%)], with bilateral hand [*n* = 173 (26.5%)] and knee [*n* = 291 (44.6%)] pain also common. After 1 month, the prevalence of pain in many anatomical regions had significantly decreased and remained relatively stable over the remainder of the study period.

Some differences were seen in the intensity and prevalence of pain by gender and age group, with female and younger people tending to report more pain. However, there were no consistent patterns (Supplementary Data S1, available at *Rheumatology Advances in Practice* online).

When considering individuals reporting taking glucocorticoids at each time point, those on glucocorticoid treatment reported similar numbers of pain sites and pain intensity as those not reporting glucocorticoid use in the first 18 months. At and after the 18-month follow-up, the intensity of pain and the proportion reporting bilateral shoulder pain was significantly higher in this group (Table 2; Supplementary Figs S1 and S2, available at *Rheumatology Advances in Practice* online), as was the intensity of pain. The proportion with widespread pain was significantly higher in those reporting glucocorticoid use at 24 months and long-term follow-up. Similar patterns were seen in reported stiffness (Supplementary Tables S1 and S2; Supplementary Figs S3–S5, available at *Rheumatology Advances in Practice* online).

**Table 2.** Pain characteristics over 2 years by current GC use status

Characteristics	Baseline	Month 1	Month 4	Month 8	Month 12	Month 18	Month 24	Long-term follow-up
GC use at given time point No GC use at given time point	*n* = 625 *n* = 17	*n* = 564 *n* = 30	*n* = 518 *n* = 33	*n* = 463 *n* = 61	*n* = 397 *n* = 94	*n* = 323 *n* = 142	*n* = 255 *n* = 181	*n* = 102 *n* = 68
Pain (0–10 NRS), median (IQR)	8 (7–9) 8 (5–8)	2 (0.75–4) 6 (2–8)	2 (1–4) 5 (3–7.5)	3 (1–5) 4 (1–6)	2 (1–4) 3 (0.75–5)	3 (1–5)^a^ 1 (0–4)	3 (1–5)^a^ 1.5 (0–4)	4 (2–6)^a^ 0.5 (0–3)
Total number of pain sites, median (IQR)	16 (10–24) 14 (5.5–20)	4 (0–10) 8 (3.25–16.25)	5 (0–10) 9 (5–16.5)	5 (0–11) 7 (0–15)	5 (0–11) 7.5 (0–14.25)	7 (0–12) 5 (0–13)	7 (0–14)^a^ 3 (0–10)	9 (0–17.75)^a^ 4 (0–9.25)
Bilateral shoulder pain	513 (82.1) 11 (64.7)	185 (32.8) 19 (63.3)	181 (34.9) 21 (63.6)	159 (34.3) 20 (32.8)	150 (37.8) 31 (33.0)	128 (39.6)^a^ 42 (29.6)	111 (43.5)^a^ 50 (27.6)	30 (44.1)^a^ 29 (28.4)
Unilateral shoulder pain	42 (6.7) – (11.8)	67 (11.9) – (13.3)	71 (13.7) 9 (9.1)	55 (11.9) 11 (18.0)	46 (11.6) 13 (13.8)	47 (14.6) 18 (12.7)	29 (11.4) 19 (10.5)	11 (16.2) 8 (7.8)
Bilateral hip pain	378 (60.5) 5 (29.4)	112 (19.9) 9 (30.0)	87 (16.8) 11 (33.3)	95 (20.5) 21 (34.4)	103 (25.9) 28 (29.8)	84 (26.0) 37 (26.1)	69 (27.1) 33 (18.2)	26 (38.2) 17 (17.7)
Unilateral hip pain	43 (6.9) 0	57 (10.1) – (3.3)	56 (10.8) – (9.1)	48 (10.4) – (3.3)	32 (8.1) 8 (8.5)	20 (6.2) 8 (5.6)	20 (7.8) 13 (7.2)	– (5.9) 10 (9.8)
Bilateral hand pain	165 (26.4) 5 (29.4)	62 (11.0) 9 (30.0)	68 (13.1) 7 (21.2)	75 (16.2) 8 (13.1)	61 (15.4) 19 (20.2)	59 (18.3) 23 (16.2)	47 (18.4) 23 (12.7)	16 (23.5) 17 (16.7)
Unilateral hand pain	55 (8.8) 0	33 (5.9) – (6.7)	34 (6.6) – (6.1)	31 (6.7) 7 (11.5)	26 (6.6) 9 (9.6)	20 (6.2) 9 (6.3)	17 (6.7) 10 (5.5)	– (4.4) 7 (6.9)
Bilateral knee pain	281 (45.0) 6 (35.3)	98 (17.4) 9 (30.0)	76 (14.7) 11 (33.3)	76 (16.4) 12 (19.7)	74 (18.6) 26 (27.7)	75 (23.2) 33 (23.2)	54 (21.2) 34 (18.8)	16 (23.5) 19 (18.6)
Unilateral knee pain	65 (10.4) – (17.7)	49 (8.7) – (6.7)	39 (7.5) – (9.1)	42 (9.1) 8 (13.1)	27 (6.8) 6 (6.4)	24 (7.4) 10 (7.0)	21 (8.2) 14 (7.7)	– (5.9) 8 (7.8)
Widespread pain	356 (57.0) 5 (29.4)	112 (19.9) 14 (46.7)	91 (17.6) 13 (39.4)	91 (19.7) 16 (26.2)	87 (21.9) 25 (26.6)	76 (23.5) 33 (23.2)	70 (27.5)^a^ 28 (15.5)	28 (41.2)^a^ 22 (21.6)

–: count supressed due to small cell count.

Values are presented as *n* (%) unless stated otherwise.

Numbers with and without GC treatment at a given time period may not total the number in Table 1 due to missing data in relation to self-reported GC use. Testing of differences in medians and proportions reporting site-specific pain in those with and without GC tested at the 12-, 18- and 24-month and long-term follow-ups (not tested before 12 months due to small numbers not reporting GC use).

a Significant difference between those reporting GC use and non-use.

### Pain and stiffness in the NorStOP study

The 652 people from the NorStOP study selected to match the age and gender distribution of the PMR cohort [mean age of 72.5 years (s.d. 8.9), 62.1% female] reported a median of 4 (IQR 0–9) sites with pain/stiffness (Table 1). The prevalence of pain and stiffness in the NorStOP study sample (Fig. 2) was generally lower across anatomical sites than in the PMR cohort at baseline. This is particularly true in the shoulders and the anterior hips, as well as the spine. The total number of painful/stiff sites and the prevalence of bilateral and widespread pain/stiffness was also considerably lower than in the PMR cohort. This was not true for unilateral pain/stiffness, where the prevalence was similar or higher in the general population than in those with PMR at the time of diagnosis.

From 1 month after PMR diagnosis, the total number of painful/stiff sites was similar in the NorStOP study sample compared with the PMR cohort, as was the prevalence of unilateral pain/stiffness in the shoulders and hands. Bilateral pain/stiffness in the shoulders and hips remained more common in the PMR cohort throughout the period than in the general population study.

## Discussion

### Principal findings

While around the time of their diagnosis, members of the PMR Cohort Study were considerably more likely to report bilateral pain in their shoulders and hips, as well as bilateral hand and knee pain, than those in the general population, this reduced quickly with glucocorticoid treatment. From the first month after PMR diagnosis, the prevalence of pain in the PMR cohort was similar to that in the general population NorStOP cohort, although bilateral shoulder and hip pain remained more common in those with PMR.

### Comparison with the literature

The locations of pain and stiffness reported by people with PMR reflect those seen in clinical practice guidelines [14]. Our study also showed high levels of pain and stiffness in the knees, wrists and hands, not areas classically associated with PMR. Detailed imaging studies using MRI and ultrasonography investigations have identified subacromial/subdeltoid bursitis and trochanteric bursitis as common PMR features, the presence of which is now included within the EULAR/ACR preliminary classification criteria [14]. Advanced imaging using PET/CT highlights that fluorodeoxyglucose (FDG) uptake adjacent to the ischial tuberosities together with involvement at the peri-articular shoulder or interspinous bursa on whole-body PET/CT is highly sensitive and specific for a diagnosis of PMR [15]. Beyond the shoulder and hip, Owen *et al.* [15, 16] also found high levels of FDG uptake in the posteromedial knee, wrist and hands, supporting likely inflammatory involvement.

Levels of pain similar to those of the general population once glucocorticoid treatment is established are in keeping with findings from the USA in which a cohort with PMR was found to have rates of opioid use, particularly chronic use, similar to those of the general population of the same age [17].

We previously showed in the PMR cohort that 40% of participants were still using glucocorticoids at the long-term follow-up. While consistent with other cohorts with PMR [4], this is considerably longer than guidelines suggest for glucocorticoid treatment in this group [18] and the reported levels of pain should be considered in the context of this ongoing glucocorticoid use in addition to other analgesics this group might be using.

### Strengths and weaknesses

The major strength of this study compared with previous studies of PMR is its recruitment from primary care, as <20% of patients in the UK with PMR ever see a rheumatology specialist and even fewer see one at the time of their diagnosis [19, 20]. By recruiting from primary care in the UK, we have included those who were diagnosed in both primary and secondary care settings, as specialists will refer patients back to primary care, where we would still identify them for the study. We ensured a high response rate at each survey stage by keeping questionnaires as short as possible and using a reminder system for non-responders. Due to our recruitment strategy, we are reliant on the GP to accurately diagnose PMR. However, we did provide all participating practices with a copy of the most recent BSR guidelines on the diagnosis of PMR [10] to improve diagnostic accuracy.

We do not have information on the pain and stiffness experience of people with PMR prior to their PMR diagnosis and glucocorticoid treatment. However, the ability to compare our cohort with a diagnosis of PMR to a general population sample does allow us to mitigate this limitation somewhat.

While we have collected detailed information on the anatomical location of pain/stiffness, we are unable to compare the severity or intensity of pain/stiffness at different sites. Additionally, participants reported any pain and stiffness on the manikins, not only that which they attributed to PMR.

### Implications for research and practice

Our finding of persistence of bilateral pain and stiffness in the shoulders and hips, at higher levels than seen in the general population, throughout the PMR Cohort Study period could arise in a number of ways. First, it could be that PMR symptoms are not well controlled for a group of people. This hypothesis is to some extent corroborated by the higher prevalence of bilateral shoulder pain throughout the study in those not reporting glucocorticoid use at the time of questionnaire completion. Second, the original presence of PMR or its treatment could have caused longer-term damage to the musculoskeletal system that has then become more prone to other non-inflammatory conditions, such as frozen shoulder and OA. Third, individuals who have experienced PMR might become more sensitized to pain in the areas affected and therefore experience, and report, more pain at these sites than the general population. In addition, the increase in unilateral pain after the time of PMR diagnosis could indicate that the pain people in the study are experiencing after their diagnosis is due, at least in part, to other conditions, rather than PMR itself (e.g. OA).

These findings suggest that careful clinical assessment and re-evaluation (including measuring inflammatory markers) is needed for people reporting persistent pain and stiffness or struggling to reduce glucocorticoids. It may be appropriate to consider specialist rheumatology referral for such people to determine whether symptoms are caused by refractory PMR or the development of RA (in which case it may be appropriate to consider DMARDs/steroid-sparing agents) or whether symptoms are caused by other non-inflammatory pathologies requiring different management. Ultrasound may be useful in people with persistent pain to differentiate between PMR and frozen shoulder [21] and guide management. Further advances in imaging, such as PET/CT, may also be useful in refractory cases, although access to these advanced imaging modalities is variable.

### Conclusions

This study showed that individuals with a primary care diagnosis of PMR report pain across a larger proportion of the body, particularly in the shoulders and hips at the time of diagnosis. However, with glucocorticoid treatment, at a population level, the anatomical distribution of pain returns quickly to that of the general population, with some residual bilateral shoulder pain.

## Supplementary Material

rkaf060_Supplementary_Data

## Data Availability

Keele University is a member of the UK Reproducibility Network and committed to the principles of the UK Concordat on Open Research Data. The School of Medicine and Keele Clinical Trials Unit have a long-standing commitment to sharing data from our studies to improve research reproducibility and to maximize benefits for patients, the wider public and the health and care system. We encourage collaboration with those who collected the data to recognize and credit their contributions. The School of Medicine and Keele Clinical Trials Unit make data available to bona fide researchers upon reasonable request via open or restricted access through a strictly controlled access procedure. The release of data may be subject to a data use agreement between the sponsor and the third party requesting the data. In the first instance, data requests and enquiries should be directed to medicine.datasharing@keele.ac.uk.
