# Supplementary material for: Patterns of pain and stiffness over 5 years in polymyalgia rheumatica: results from the PMR Cohort Study
Source: Rheumatol Adv Pract. 2025 Jun 5;9(3):rkaf060. doi: 10.1093/rap/rkaf060 (PMC12204186; doi:10.1093/rap/rkaf060)

Supplementary Table 1: Stiffness characteristics over 2 years (n (%) unless otherwise stated)

| Characteristic | Baseline  (n=650) | Month 1  (n=599) | Month 4  (n=554) | Month 8  (n=527) | Month 12  (n=495) | Month 18  (n=470) | Month 24  (n=445) | Long-term follow-up  (n=197) |
| --- | --- | --- | --- | --- | --- | --- | --- | --- |
| Stiffness (0-10 NRS) (Median IQR) | 8 (7,9)^a^ | 2 (1, 5)^a b^ | 3 (1, 5)^a b^ | 3 (1, 5)^a b^ | 3 (1, 5)^a b^ | 3 (1, 5)^a b^ | 3 (1, 5) ^b^ | 3 (0, 5)^b^ |
| Total number of stiff sites (Median (IQR)) | 12 (6, 20)^a b^ | 2 (0, 8)^a b^ | 4 (0, 9)^a b^ | 4 (0, 10)^a b^ | 4 (0, 10)^a b^ | 4.5 (0, 11)^a b^ | 4 (0, 11)^a b^ | 3 (0, 9)^b^ |
| Bilateral shoulder stiffness | 427 (65.5) | 146 (24.3)^b^ | 163 (29.4)^a b^ | 164 (31.1)^b^ | 163 (32.8)^b^ | 153 (32.4)^b^ | 146 (32.7)^b^ | 47 (23.9)^b^ |
| Unilateral shoulder stiffness | 47 (7.2) | 53 (8.8) | 59 (10.6) | 483 (8.5) | 30 (6.0) | 39 (8.3) | 38 (8.5) | 17 (8.6) |
| Bilateral hip stiffness | 313 (48.0)^b^ | 106 (17.6)^b^ | 98 (17.7)^a b^ | 123 (23.3)^a b^ | 112 (22.5)^a b^ | 111 (23.5)^a b^ | 107 (24.0)^b^ | 38 (19.3) |
| Unilateral hip stiffness | 43 (6.6) | 33 (5.5) | 30 (5.4)^a^ | 25 (4.7)^a^ | 23 (4.6) | 25 (5.3) | 25 (5.6) | 12 (6.1) |
| Bilateral hand stiffness | 130 (19.9) | 68 (11.3) | 72 (13.0) | 80 (15.2)^a b^ | 64 (12.9)^a^ | 68 (14.4) | 54 (12.1) | 32 (16.2) |
| Unilateral hand stiffness | 42 (6.4) | 22 (3.7) | 22 (4.0) | 31 (5.9) | 26 (5.2) | 21 (4.5) | 18 (4.0) | 10 (5.1) |
| Bilateral knee stiffness | 267 (41.0)^b^ | 108 (18.0)^a b^ | 93 (16.8)^a b^ | 126 (23.9)^a b^ | 106 (21.3)^a^ | 121 (25.6)^a b^ | 111 (24.9) | 37 (18.8) |
| Unilateral knee stiffness | 47 (7.2) | 35 (5.8) | 32 (5.8) | 21 (4.0) | 22 (4.4) | 24 (5.1) | 19 (4.3) | 7 (3.6) |
| Widespread stiffness | 291 (44.6)^b^ | 86 (14.3)^a b^ | 98 (17.7)^a b^ | 106 (20.1)^a b^ | 88 (17.7)^a b^ | 97 (20.6)^a b^ | 88 (19.7)^b^ | 37 (18.8)^b^ |

^a^ Significant difference between males and females; ^b^ Significant difference between age groups (<60, 60-69, 70-79, ≥80 years)

Supplementary Table 2: Stiffness characteristics over 2 years (n (%) unless otherwise stated) in those using GCs at each time point

| Characteristic | Baseline | Month 1 | Month 4 | Month 8 | Month 12 | Month 18 | Month 24 | Long-term follow-up |
| --- | --- | --- | --- | --- | --- | --- | --- | --- |
| **GC use at given time point**  **No GC use at given time point** | (n=625)  (n=17) | (n=564) (n=30) | (n=518) (n=33) | (n=463) (n=61) | (n=397) (n=94) | (n=323) (n=142) | (n=255) (n=181) | (n=102) (n=68) |
| Stiffness (0-10 NRS) (Median IQR) | 8 (7, 9)  8 (5, 8) | 2 (0.75, 4)  5 (2, 8) | 2 (1, 4)  5 (3, 7.5) | 3 (1, 5)  4 (1, 6) | 2 (1, 4)  3 (0.75, 5) | 3 (1, 5)^a^  1 (0, 4) | 3 (1, 5)^a^  1.5 (0, 4) | 4 (2, 6)^a^  0.5 (0, 3) |
| Total number of stiff sites (Mean SD) | 12 (6, 20)  9 (4, 15.5) | 2 (0, 8)  7 (0, 16) | 4 (0, 8.25)  8 (4, 11) | 4 (0, 10)  5 (0,12) | 4 (0, 10)  4 (0, 12) | 6 (0, 10)^a^  6 (0, 12) | 6 (0, 12)^a^  1 (0, 10) | 0 (0, 8)^a^  6 (0, 11) |
| Bilateral shoulder stiffness | 414 (66.2)  9 (52.9) | 132 (23.4)  13 (43.3) | 145 (28.0)  15 (45.5) | 137 (29.6)  25 (41.0) | 137 (34.5)  25 (26.6) | 122 (37.8)^a^  27 (19.0) | 98 (38.4)^a^  44 (24.3) | 22 (32.4)  23 (22.6) |
| Unilateral shoulder stiffness | 45 (7.2)  - (11.8) | 48 (8.5)  - (10.0) | 55 (10.6)  3 (9.1) | 41 (8.9)  - (6.6) | 20 (5.0)  8 (8.5) | 23 (7.1)  16 (11.3) | 22 (8.6)  16 (8.8) | 6 (8.8)  - (3.9) |
| Bilateral hip stiffness | 307 (49.1)  - (23.5) | 99 (17.6)  5 (16.7) | 89 (17.2)  7 (21.2) | 99 (21.4)  24 (39.3) | 91 (22.9)  21 (22.3) | 82 (25.4)  26 (18.3) | 69 (27.1)  35 (19.3) | 24 (35.3)^a^  12 (11.8) |
| Unilateral hip stiffness | 43 (6.9)  0 | 31 (5.5)  - (3.3) | 28 (5.4)  2 (6.1) | 23 (5.0)  - (3.3) | 19 (4.8)  - (4.3) | 20 (3.2)  5 (3.5) | 19 (7.5)  6 (3.3) | - (2.9)  7 (6.9) |
| Bilateral hand stiffness | 124 (19.8)  - (17.7) | 60 (10.6)  7 (23.3) | 62 (12.0)  8 (24.2) | 68 (14.7)  12 (19.7) | 48 (12.1)  15 (16.0) | 47 (14.6)  19 (13.4) | 34 (13.3)  18 (9.9) | 13 (19.1)  11 (10.8) |
| Unilateral hand stiffness | 40 (6.4)  - (5.9) | 21 (3.7)  - (3.3) | 22 (4.3)  0 | 27 (5.8)  - (6.6) | 20 (5.0)  5 (5.3) | 15 (4.6)  6 (4.2) | 9 (3.5)  9 (5.0) | - (2.9)  5 (4.9) |
| Bilateral knee stiffness | 256 (41.0)  17 (41.2) | 97 (17.2)  9 (30.0) | 79 (15.3)  12 (36.4) | 108 (23.3)  17 (27.9) | 78 (19.7)  27 (28.7) | 85 (26.3)  32 (22.5) | 65 (25.5)  43 (23.8) | 16 (23.5)  18 (17.7) |
| Unilateral knee stiffness | 45 (7.2)  - (11.8) | 34 (6.0)  - (3.3) | 32 (6.2)  0 | 19 (4.1)  - (3.3) | 19 (4.8)  - (3.2) | 22 (6.8) ^a^  - (1.4) | 14 (5.5)  5 (2.8) | 0  - (2.9) |
| Widespread stiffness | 283 (45.3)  6 (35.3) | 75 (13.3)  10 (33.3) | 89 (17.2)  7 (21.2) | 89 (19.2)  16 (16.2) | 68 (17.1)  19 (20.2) | 72 (22.3)  23 (16.2) | 60 (23.5)^a^  26 (14.4) | 19 (27.9) ^a^  15 (14.7) |

Note: numbers with and without GC treatment at a given time period may not total the number in Supplementary Table 1 due to missing data in relation to self-reported GC use. Testing of differences in medians and proportions reporting site-specific pain in those with and without GC tested at 12-, 18-, 24-month and long-term follow-ups (not tested before 12 months due to small numbers not reporting GC use). ^a^Significant difference between those reporting GC use and non-use. – count supressed due to small cell count


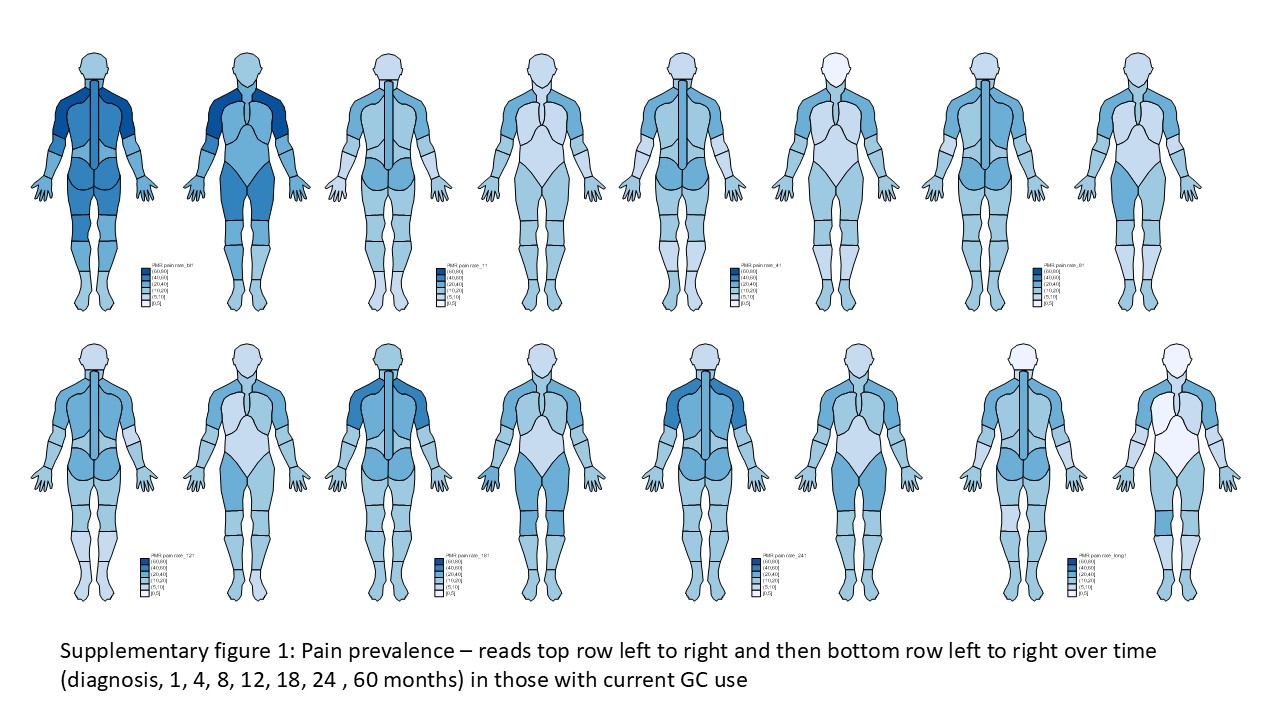


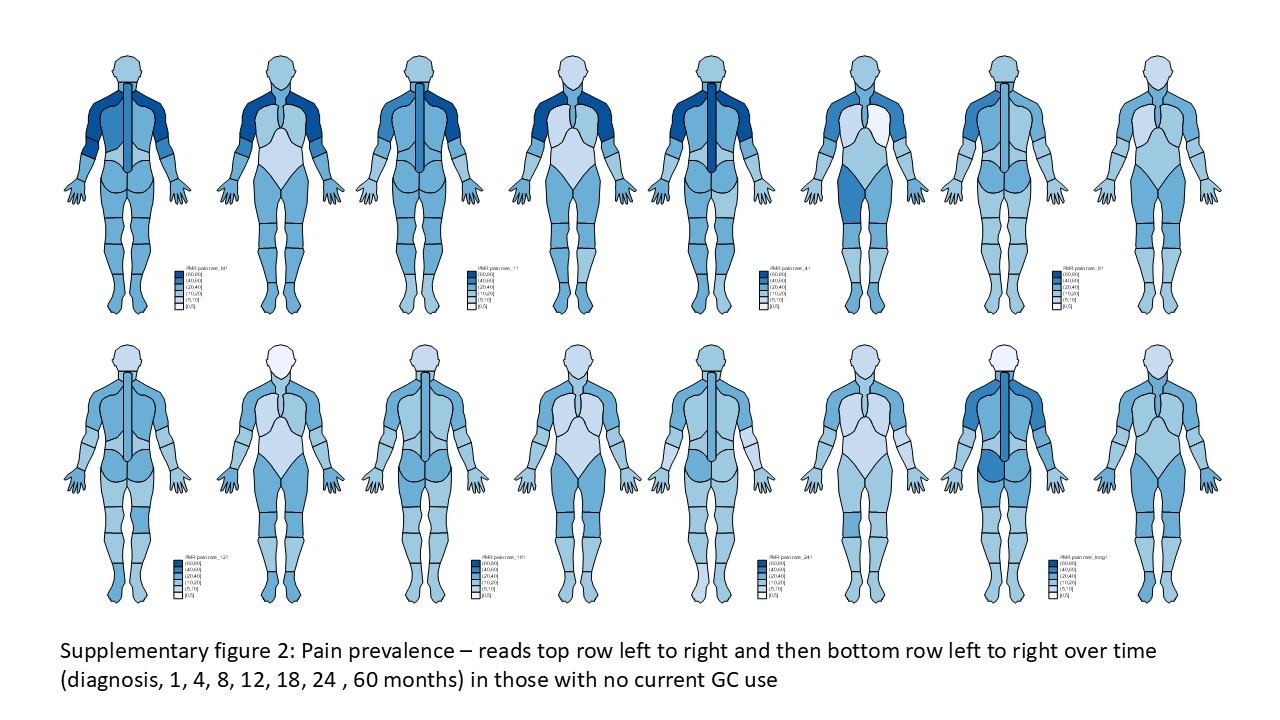


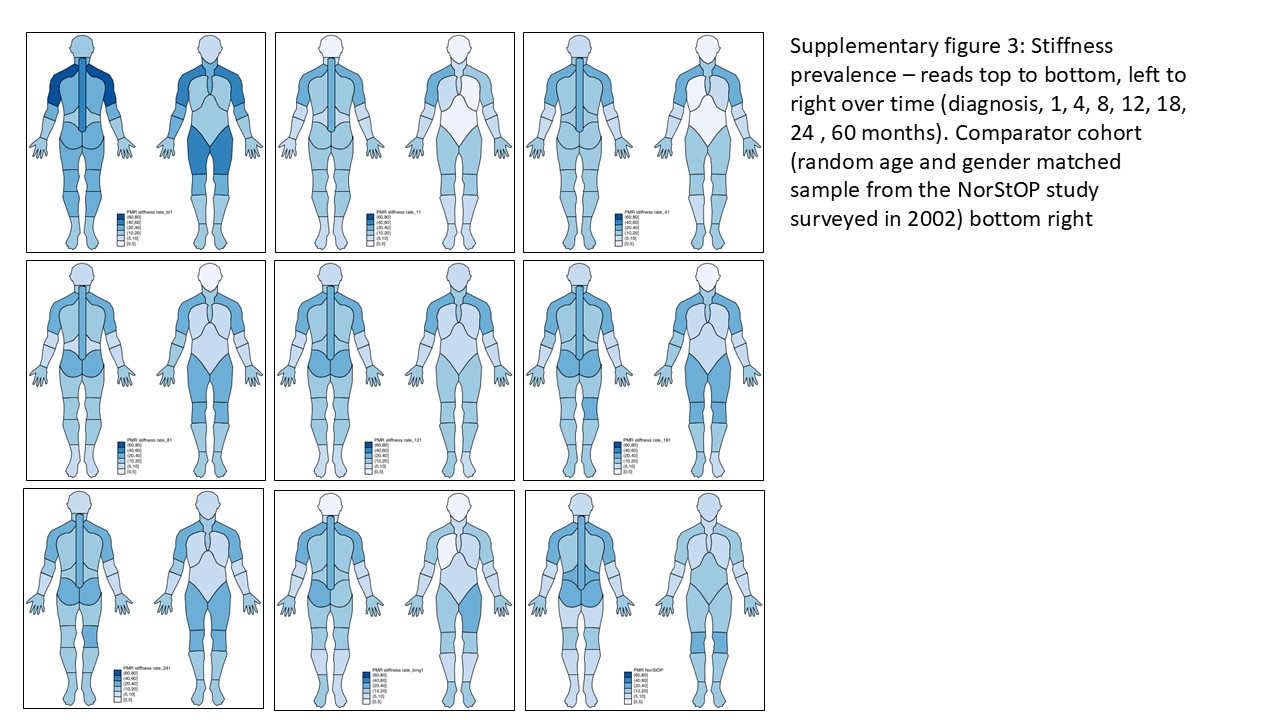


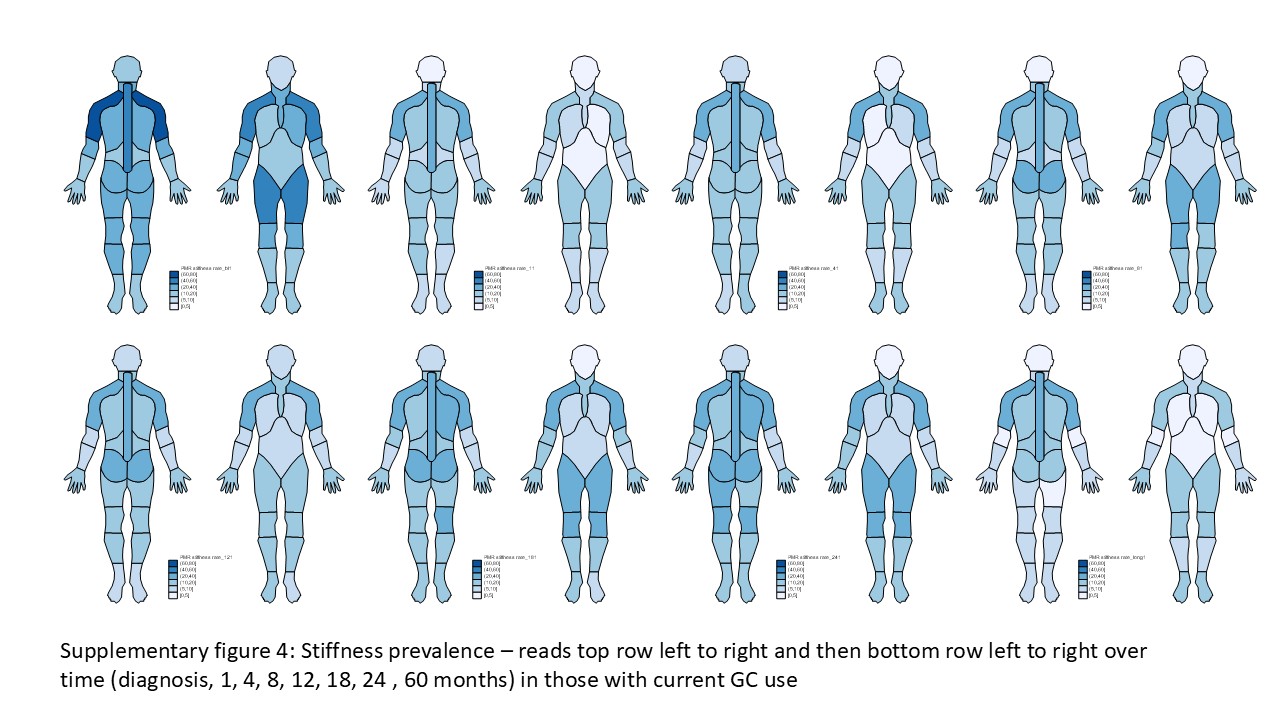


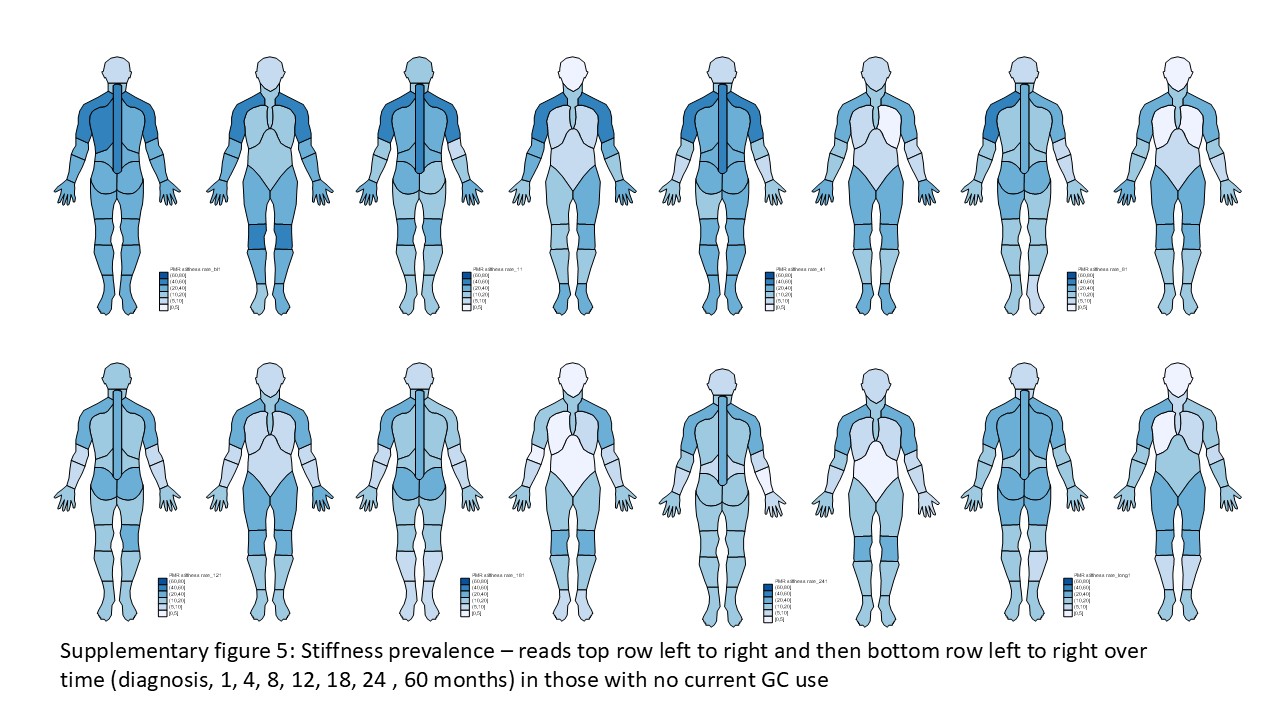

Supplement: rkaf060_Supplementary_Data [file rkaf060_supplementary_data.zip › 24-283 Supplementary Tables and Figures.docx]
